# A Long Road to Safer Food

**DOI:** 10.3390/toxins12070453

**Published:** 2020-07-14

**Authors:** Filippo Rossi

**Affiliations:** Section of Food Science and Nutrition, Department of Animal Sciences, Food and Nutrition, Faculty of Agricultural, Food and Environmental Sciences, Catholic University of Sacred Heart, Via Emilia Parmense 84, 29122 Piacenza, Italy; filippo.rossi@unicatt.it

As a side effect of food production, mycotoxins have always accompanied humanity, even if the danger posed by these molecules has only recently been understood and new research has begun to identify and study ways to reduce their presence in food.

This Special Issue of *Toxins* includes papers on new findings concerning well-known mycotoxins, results of studies regarding emerging mycotoxins, such as alternaria and botryodiplodin, and new techniques to reduce mycotoxin contamination in processed cereals.

Reliable data on the presence of mycotoxins in food is very important in the toxicological evaluation of the risk associated with these toxic fungal compounds. Two papers cover this subject: Quevedo-Garza et al. [1] analyze Mexican infant formula food for aflatoxin M1 and Zentai et al. [2] determine the fumonisins in Hungarian maize-based food. 

*Fusarium* spp., together with *Aspergillus* spp., are the most relevant fungi genus responsible for mycotoxin production. Researchers have focused their attention on cereals, while neglecting other crops. A paper from a Chinese group reports on the identification of the *Fusarium* species causing sweet pepper fruit rot and on the kinds of mycotoxins produced by these microorganisms [3].

A new toxic molecule produced by a fungal parasite of soybean is the focus of two papers from Abbas et al. who investigate the production of botryodiplodin [4] and its toxicity [5], while another contribution [6] considers secalonic acids, which are the main ergot ergochromes in overall ergot toxicity.

We have observed not only the appearance of new or emerging mycotoxins, but also of new foods, such as insects, that can also be contaminated by mycotoxins. On this topic, a paper in this Special Issue studies the metabolism of aflatoxin B1 in the larvae of the black soldier fly (*Hermetia illucens*) [7].

The reduction of mycotoxin contamination can be obtained by intervening during the cultivation or storage of products. Research carried out by Giorni et al. [8] tested the efficacy of the fungicide azoxystrobin on fungal parasites of rice and obtained a strong reduction (−67%) of sterigmatocystin while deoxynivalenol remained unaffected. 

A clear reduction in *Fusarium*-produced toxins can be observed in the paper of Brodal et al. [9], by sieving oat grains and removing broken kernels, which are more contaminated than intact ones.

The last research article of the Special Issue describes an analytical method for the detection of 19 mycotoxins and three phytoestrogens in fish feed and fish meat [10]. The reduction of the risk posed to human health by mycotoxins requires the development and validation of reliable methods to monitor mycotoxins in feed and food.

The three reviews included in the Special Issue cover as many topics. Issues related to the use of lactic acid bacteria as aflatoxin binders in developing countries are discussed in the review of Ahlberg et al [11]. Kamle et al. [12] summarize the effect of fumonisin on human health and the strategies to reduce the level of this toxin in food. A group of emerging mycotoxins, those produced by *Alternaria*, is the focus of Crudo et al [13], who analyze “the most relevant data concerning the occurrence and toxicity of mycotoxins produced by *Alternaria* spp., (….) alone or in combination with other mycotoxins and bioactive food constituents”.

In conclusion, all the contributions to this Special Issue expand our current knowledge and, as Guest Editor, I am happy and proud to present this issue to the community of scientists involved in research on mycotoxins.

All research and review articles proposing novelties and overviews, respectively, were successfully and carefully selected for this Special Issue after rigorous revision by the expert peer reviewers. As the Guest Editor, I would like to express my deep appreciation to all the selfless and fair reviewers.
